# *Salmonella enterica* Serovar Typhimurium Strategies for Host Adaptation

**DOI:** 10.3389/fmicb.2017.01983

**Published:** 2017-10-12

**Authors:** Christopher J. Anderson, Melissa M. Kendall

**Affiliations:** Department of Microbiology, Immunology, and Cancer Biology, University of Virginia School of Medicine,, Charlottesville, VA, United States

**Keywords:** *Salmonella*, macrophages, signaling pathways, infection, microbiota

## Abstract

Bacterial pathogens must sense and respond to newly encountered host environments to regulate the expression of critical virulence factors that allow for niche adaptation and successful colonization. Among bacterial pathogens, non-typhoidal serovars of *Salmonella enterica*, such as serovar Typhimurium (*S.* Tm), are a primary cause of foodborne illnesses that lead to hospitalizations and deaths worldwide. *S*. Tm causes acute inflammatory diarrhea that can progress to invasive systemic disease in susceptible patients. The gastrointestinal tract and intramacrophage environments are two critically important niches during *S*. Tm infection, and each presents unique challenges to limit *S*. Tm growth. The intestinal tract is home to billions of commensal microbes, termed the microbiota, which limits the amount of available nutrients for invading pathogens such as *S.* Tm. Therefore, *S.* Tm encodes strategies to manipulate the commensal population and side-step this nutritional competition. During subsequent stages of disease, *S.* Tm resists host immune cell mechanisms of killing. Host cells use antimicrobial peptides, acidification of vacuoles, and nutrient limitation to kill phagocytosed microbes, and yet *S.* Tm is able to subvert these defense systems. In this review, we discuss recently described molecular mechanisms that *S.* Tm uses to outcompete the resident microbiota within the gastrointestinal tract. *S.* Tm directly eliminates close competitors via bacterial cell-to-cell contact as well as by stimulating a host immune response to eliminate specific members of the microbiota. Additionally, *S.* Tm tightly regulates the expression of key virulence factors that enable *S.* Tm to withstand host immune defenses within macrophages. Additionally, we highlight the chemical and physical signals that *S*. Tm senses as cues to adapt to each of these environments. These strategies ultimately allow *S.* Tm to successfully adapt to these two disparate host environments. It is critical to better understand bacterial adaptation strategies because disruption of these pathways and mechanisms, especially those shared by multiple pathogens, may provide novel therapeutic intervention strategies.

## *Salmonella enterica* Serovar Typhimurium Infection

Non-typhoidal serovars of *Salmonella enterica* (NTS) are leading causes of foodborne illness and diarrheal disease worldwide (Graham et al., 2000; Vojdani et al., 2008; Scallan et al., 2011; Ansari et al., 2012; Kabir et al., 2012; Kozak et al., 2013). In the United States, NTS infections result in more hospitalizations and deaths compared to infections caused by any other foodborne pathogen (Scallan et al., 2011). Among NTS, serovar Typhimurium (*S.* Tm) is one of the most commonly isolated from patients around the globe (Galanis et al., 2006). NTS infections typically present as a self-limiting diarrheal disease (Acheson and Hohmann, 2001; Gordon, 2008); however, NTS gastrointestinal infections can develop into systemic disease in immunocompromised patients, as well as a small subset of immunocompetent patients (Acheson and Hohmann, 2001; Gordon, 2008). Currently, there are no effective vaccines against gastrointestinal infections. Additionally, treatment options are limited because antibiotics may lead to increased levels of *S.* Tm shedding and also because *S.* Tm is developing resistance to many antibiotics (Wiström et al., 1992; Martin, 2012; Diard et al., 2014; Gopinath et al., 2014; Strugnell et al., 2014). Accordingly, alternative therapeutic intervention strategies are needed.

*S.* Tm establishes infection in the gastrointestinal tract and causes acute gastroenteritis. A common feature of *S.* Tm disease is inflammatory diarrhea indicated by the presence of neutrophils in patient stool samples (Harris et al., 1972). Type III secretion systems (T3SSs) are molecular syringe-like structures that allow Gram-negative organisms to directly inject effector proteins into the cytosol of host cells (Deng et al., 2017). *S.* Tm uses a T3SS encoded within *Salmonella* Pathogenicity Island (SPI) 1 (T3SS-1) to actively invade epithelial cells, induce inflammation, and breach the epithelial barrier (Galán and Curtiss, 1989; Tsolis et al., 1999). After exiting the intestinal tract, *S.* Tm is phagocytosed by resident and recruited immune cells, including macrophages. *S.* Tm utilizes the SPI-2 encoded T3SS (T3SS-2) to survive and replicate within these phagocytes (Hensel et al., 1995; Ochman et al., 1996; Shea et al., 1996). The cumulative effects of T3SS-2 cause unchecked bacterial replication during systemic infection and lethal disease (Yoon et al., 2009).

Decades of research has identified a vast repertoire of *S.* Tm virulence determinants (extensively reviewed in Fàbrega and Vila, 2013). Recent studies have expanded our understanding of factors that influence virulence gene expression, including growth phase and environmental signals (Kröger et al., 2013; Srikumar et al., 2015); however, less is known about the signal transduction pathways that link environmental signals to virulence gene expression. In this review, we discuss recent findings concerning strategies that *S*. Tm uses to overcome microbiota- and host-derived obstacles within the intestinal and intramacrophage environments. Additionally, we highlight signals that *S.* Tm uses to coordinate expression of virulence genes required for adaptation to these distinct environments.

## Making Room Within the Crowded Intestinal Tract

The gastrointestinal tract is home to billions of microbes termed the microbiota. Interactions between the host, the microbiota, and pathogens have profound impacts on infection (Yurist-Doutsch et al., 2014; McKenney and Pamer, 2015; Bäumler and Sperandio, 2016; Gart et al., 2016; Kendall and Sperandio, 2016; McKenney et al., 2016). The microbiota function as a barrier to limit pathogen colonization and shedding (Endt et al., 2010), an ability collectively referred to as colonization resistance. Colonization resistance is largely attributed to the ability of the microbiota to outcompete invading pathogens for nutrients; however, the microbiota can also modulate host mucosal immune responses important for clearing infection (Endt et al., 2010; Thiemann et al., 2017). Dysbiosis, or alterations to the microbiota, creates a non-competitive niche in which *S.* Tm is able to establish infection and rapidly replicate in the intestine. Antibiotic use results in disruptions to the microbiota and is a key risk factor associated with the *Salmonella*-associated diarrhea (Acheson and Hohmann, 2001; Gordon, 2008).

**FIGURE 1:**
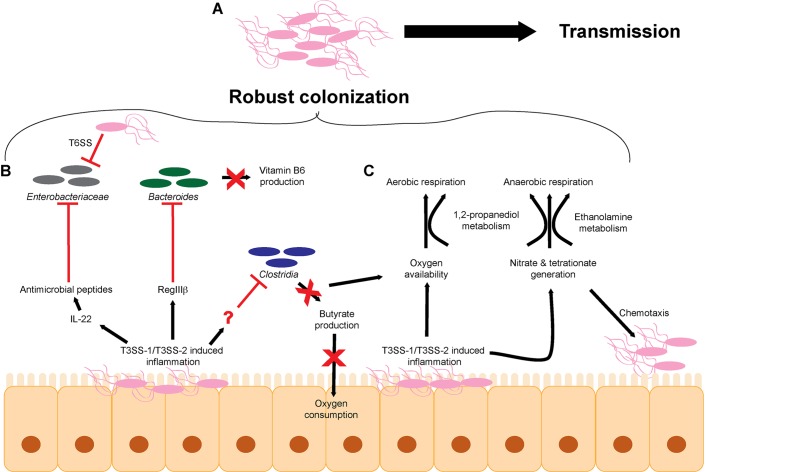
*S.* Tm overcomes microbiota-mediated colonization resistance in the intestinal tract. **(A)** The cumulative effects of T3SS-1/T3SS-2-induced inflammation lead to robust colonization of the intestinal tract and efficient transmission to a new host. **(B)** *S.* Tm utilizes the T3SS-1 and T3SS-2 to generate a robust host immune response within the intestinal tract to eliminate members of the microbiota. **(C)** T3SS-1/T3SS-2-induced inflammation leads to the production of metabolites such as electron acceptors including oxygen, nitrate, and tetrathionate that promote aerobic and anaerobic respiration. T3SS, type III secretion system; T6SS, type VI secretion system.

Although antibiotic-related dysbiosis provides an opening for *S.* Tm to establish infection, *S*. Tm also directly perturbs the microbiota to enhance and prolong infection (**Figure 1A**). *S.* Tm relies primarily on the T3SS-1, and to an extent the T3SS-2, to induce host inflammation, and the resulting innate immune response non-specifically targets the microbiota along with *S*. Tm (Barthel et al., 2003; Coburn et al., 2005; Stecher et al., 2007; Barman et al., 2008; Lawley et al., 2008; Sekirov et al., 2008; Juricova et al., 2013; Lam and Monack, 2014; Drumo et al., 2016; Rivera-Chávez et al., 2016b). As a result of T3SS-induced inflammation, a proportion of infecting *S.* Tm cells succumb to host immune responses; however, a sufficient amount of *S.* Tm cells survive to successfully establish infection (Raffatellu et al., 2009; Liu et al., 2012; Bogomolnaya et al., 2013; Maier et al., 2014; Diaz-Ochoa et al., 2016). Several environmental conditions contribute to T3SS-1 expression (Golubeva et al., 2012). For example, oxygen limitation and high salt concentrations enhance SPI-1 expression and epithelial cell invasion (Galán and Curtiss, 1990; Lee and Falkow, 1990; Bajaj et al., 1996; Mizusaki et al., 2008), whereas bile and some long and short chain fatty acids, such as a butyrate, oleate, myristate, and palmitate, repress T3SS-1 expression (Prouty and Gunn, 2000; Lawhon et al., 2002; Gantois et al., 2006; Eade et al., 2016; Golubeva et al., 2016). The balance of activating and repressing signals is thought to enrich *S.* Tm invasion in the ileum *in vivo* (Lawhon et al., 2002; Gantois et al., 2006; Eade et al., 2016). Expression of the T3SS-1 is regulated by a feed-forward loop in which the regulatory proteins HilD, HilC, and RtsA positively control expression of the master transcription factor HilA (Bajaj et al., 1995; Ellermeier et al., 2005). HilD, HilC, and RtsA are transcription factors that bind to the *hilA* promoter to induce *hilA* expression (Schechter and Lee, 2001; Olekhnovich and Kadner, 2002; Ellermeier and Slauch, 2004). HilA then activates the expression of the remaining transcription factors and structural components of the T3SS-1, as well as non-SPI-1-encoded effectors (Phoebe Lostroh and Lee, 2001). Expression of the T3SS-1 core regulatory system is in turn regulated by accessory factors that are presumed to respond to environmental signals (Golubeva et al., 2012), but how these signals are incorporated into the SPI-1 regulatory pathway has not been fully elucidated.

Besides causing unspecific disruption to the microbiota as a whole, *S.* Tm-induced inflammation impacts particular intestinal microbes that alter concentrations of metabolites and/or host responses that would otherwise limit *S*. Tm infection (**Figure 1B**). For example, *S.* Tm infection induces RegIIIβ expression, and RegIIIβ is directly bactericidal against *Bacteroides* sp. and *Eubacterium rectale* (Miki et al., 2017). RegIII lectin family proteins are expressed in the intestinal tract and are important for maintaining intestinal homeostasis and combating pathogens (Cash et al., 2006; Vaishnava et al., 2008; Miki et al., 2012). Suppression of *Bacteroides* sp. is associated with changes in metabolite availability in the gut, most notably a decrease in vitamin B6 concentrations (Miki et al., 2017). Significantly, reconstitution of *Bacteroides* or supplementation of vitamin B6 contributes to the resolution of *S.* Tm infection, further underscoring the complex interplay of host, bacteria, and metabolites (Miki et al., 2017). *S.* Tm T3SS-1 and T3SS-2-induced inflammation also leads to depletion of *Clostridia* sp. in the intestine, which enhances *S.* Tm colonization (Rivera-Chávez et al., 2016b) (detailed in the next section). The host factor that directly depletes *Clostridia* sp. has not been identified.

Additionally, commensal *Escherichia coli* represents a minor component of the microbiota during homeostasis, but during general dysbiosis, the proportion of *E. coli* within the bacterial community increases (Winter et al., 2013). *E. coli* and *S*. Tm compete for metabolites and deploy molecules to limit growth of each other (Raffatellu et al., 2009; Deriu et al., 2013; Sassone-Corsi et al., 2016). For example, iron is an essential nutrient for most microbes and is typically limited within the host; therefore, microbes have evolved mechanisms to scavenge iron (Behnsen and Raffatellu, 2016). *E. coli* and *S*. Tm produce and secrete siderophores, which are small molecules that chelate iron (Crouch et al., 2008; Behnsen and Raffatellu, 2016). *E. coli* siderophores can be conjugated to microcins, small antibacterial peptides that kill bacterial cells through an unknown mechanism (Rebuffat, 2012; Sassone-Corsi et al., 2016). To fight back, the *S.* Tm T3SS-1 and T3SS-2 promote expression of the chemokine IL-22, which results in the production of antimicrobials that kill *E. coli* but are ineffective against *S.* Tm (Godinez et al., 2008; Raffatellu et al., 2009; Stelter et al., 2011; Liu et al., 2012; Behnsen et al., 2014). By inducing IL-22, *S*. Tm eliminates Enterobacteriaceae species and thus direct nutritional competitors.

*S.* Tm also takes direct action against competitors using a type VI secretion system (T6SS). T6SSs are commonly found in the Proteobacteria and Bacteroidetes phyla and are structurally homologous to bacteriophage tail complexes (Bönemann et al., 2009; Russell et al., 2014; Hood et al., 2017). These dynamic structures contract to directly inject effector proteins into target cells (Basler et al., 2012). Although T6SS can inject effector proteins into host cells (Ma and Mekalanos, 2010; Schwarz et al., 2010), increasing evidence suggest that T6SSs primarily target and cause subsequent death of bacterial cells (Mougous et al., 2006; Pukatzki et al., 2006; Bingle et al., 2008; Cianfanelli et al., 2016). The T6SS encoded by *S.* Tm has selective bactericidal efficacy against commensal organisms *in vitro*, including other members of the Enterobacteriaceae, such as *E. coli* W3110, *Klebsiella oxytoca*, and *Klebsiella variicola* (Brunet et al., 2015; Sana et al., 2016). However, *Enterobacter cloacae* and *E. coli* JB2 are resistant to *S.* Tm T6SS attack (Sana et al., 2016). It is currently unclear why different species are susceptible or resistant. The efficacy of the *S*. Tm T6SS against *K. oxytoca* data were validated *in vivo* during intestinal co-infection studies (Sana et al., 2016). These data support an important role for the *S*. Tm T6SS against other members of the microbiota; however, the benefits of selective killing remain to be defined. A better understanding of the commensal organisms that are directly targeted by *S.* Tm via the T6SS *in vivo* may reveal essential metabolites that are being differentially regulated that may impact *S.* Tm growth or virulence gene expression.

Collectively, these findings reveal that a central strategy *S.* Tm uses to colonize the host is to actively displace members of the microbiota. *S*. Tm achieves this through manipulation and exploitation of host responses (Stecher et al., 2007; Mooney et al., 2015). Moreover, *S*. Tm is capable of directly eliminating particular bacterial species. This transforms the intestinal tract into an environment that *S.* Tm is optimized to survive in and ultimately lowers the barrier of colonization resistance.

## Metabolism in the Face of Inflammation

Dysbiosis not only results in changes in the bacterial populations but also changes the chemistry of the intestine (Zeng et al., 2017). Hence, a second aspect of *S.* Tm infection includes exploiting nutrients generated specifically during infection. The majority of bacteria that comprise the microbiota are obligate anaerobes that rely on fermentation for growth (Gibson and Roberfroid, 1995). Enterobacteriaceae, including *S.* Tm, are able to gain energy by respiration. Respiration generates higher amounts of ATP compared to fermentation and thereby enables *S*. Tm to outgrow many members of the microbiota (Wiles et al., 2006; Brochier-Armanet et al., 2009; Marteyn et al., 2010; Maier et al., 2013; Ng et al., 2013; Winter et al., 2013; Zeng et al., 2017) (**Figure 1C**). During homeostasis, host colonocytes consume oxygen, yielding a localized environment characterized by low oxygen partial pressure (Carreau et al., 2011; Espey, 2013). Colonocytes preferentially oxidize short-chain fatty acids, such as butyrate, to respire oxygen (Hamer et al., 2008; Donohoe et al., 2012; Kelly et al., 2015). Because *Clostridia* are the major producers of butyrate in the intestine, depletion of *Clostridia* with antibiotics or during *S*. Tm infection leads to a concomitant decrease in oxygen consumption by colonocytes (Sekirov et al., 2008; Louis and Flint, 2009; Gill et al., 2012; Vital et al., 2014; Rivera-Chávez et al., 2016b). *S.* Tm capitalizes on newly available oxygen to rapidly grow within the intestine (Rivera-Chávez et al., 2016b). These findings reveal a complex role for oxygen during infection. On the one hand, low levels of oxygen enhance SPI-1 expression, leading to increased inflammation and subsequent restriction of commensal organisms such as *Clostridia*. On the other hand, newly available oxygen promotes growth within the intestine. Oxygen limitation might therefore allow *S.* Tm to regulate the kinetics of SPI-1 expression within the intestine. In this proposed model, oxygen limitation early on during infection would promote SPI-1 expression, which would then in turn deplete commensal *Clostridia* via the host immune response, which would then result in an increase in oxygen availability leading to *S.* Tm outgrowth and subsequent reduction in SPI-1 expression. Additionally, the decrease in butyrate availability may also restrict the abundance of butyrate metabolizing members of the microbiota leading to even greater levels of dysbiosis.

The electron acceptors nitrate and tetrathionate also support *S*. Tm growth during infection (Winter et al., 2010; Lopez et al., 2012, 2015). The T3SS-1-secreted effector SopE is a bacteriophage-encoded activator of host Rho GTPases that results in host cytoskeletal rearrangements and activation of immune signaling pathways (Hardt et al., 1998). Additionally, SopE induces expression of host inducible nitric oxide synthase (iNOS) and generates nitrate (Lopez et al., 2012). Tetrathionate generation is a two-step process. Microbiota-produced hydrogen sulfide is converted by host colonocytes to thiosulfate, which reacts with oxygen radicals produced by host NADPH oxidase to generate tetrathionate (Winter et al., 2010). Tetrathionate reduction is coupled to ethanolamine, 1,2-propanediol, or fructose-asparagine oxidation (Price-Carter et al., 2001; Thiennimitr et al., 2011; Ali et al., 2014; Sabag-Daigle et al., 2016; Faber et al., 2017). Ethanolamine is a component of phosphatidylethanolamine, one of the most abundant phospholipids in host and microbial membranes (Randle et al., 1969; Dawaliby et al., 2015). The turnover of enterocytes and microbial cells as well as the diet provide a continuously replenished source of ethanolamine in the intestine (Cotton, 1972; Kawai et al., 1974; Dowhan, 1997, 2003; Snoeck et al., 2005; Bakovic et al., 2007). The accumulation of 1,2-propanediol depends primarily on the microbiota. It is thought that the ability of *Bacteroides* sp. to breakdown complex carbohydrates allows for the production of 1,2-propanediol as a byproduct of fermentation of methyl-pentoses (Faber et al., 2017), although experiments are needed to demonstrate this during the course of infection. This model is supported by the findings that 1,2-propanediol is nearly undetectable in germ-free mice; however, the presence of either *Bacteroides fragilis* or *Bacteroides thetaiotaomicron* is associated with 1,2-propanediol accumulation (Faber et al., 2017). Additionally, expression of *S*. Tm genes coding for 1,2-propanediol metabolism are induced in the presence of *B. thetaiotaomicron in vivo* (Ng et al., 2013; Faber et al., 2017). Furthermore, the presence of *B. thetaiotaomicron* within the intestinal tract enhances *S.* Tm expression of additional carbohydrate metabolism and transport genes, including sialic acid and fucose catabolic pathways (Ng et al., 2013). The *B. thetaiotaomicron*-encoded sialidase liberates sialic acid, which promotes *S.* Tm sialic acid metabolism and intestinal growth (Ng et al., 2013). These data indicate a somewhat paradoxical relationship between *S.* Tm and *Bacteroides* sp. As discussed above, *S.* Tm-induced inflammation restricts the levels of *Bacteroides* sp. (Miki et al., 2017) and yet *Bacteroides* sp. are critical for the accumulation of multiple metabolites that *S*. Tm utilizes during infection (Ng et al., 2013; Faber et al., 2017). These seemingly conflicting ideas suggest an even more complex relationship between pathogen and microbiota. Rather than presence or absence of commensals, *S.* Tm may require a fine-tuned abundance of microbes to generate beneficial metabolites without these organisms directly competing with *S.* Tm.

*S.* Tm also exploits electron acceptors as spatiotemporal cues for colonization and tissue invasion. Nitrate and tetrathionate are indirect signals for *S.* Tm chemotaxis and thereby influence subsequent stages in *S*. Tm infection (Rivera-Chavez et al., 2013). The chemotaxis proteins Aer and Tsr sense changes in redox and proton motive force during tetrathionate and nitrate respiration, respectively (Edwards et al., 2006; Rivera-Chávez et al., 2016a). Both Aer and Tsr promote T3SS-1/T3SS-2-dependent intestinal colonization (Rivera-Chavez et al., 2013). Tsr-dependent chemotaxis correlates with localized host production of iNOS and enables *S.* Tm to invade ileal Peyer’s patches (Rivera-Chávez et al., 2016a). Surprisingly, no additive effect is seen with Aer and Tsr-dependent chemotaxis (Rivera-Chavez et al., 2013). This suggests that these two sensing pathways may be interconnected and perhaps functionally redundant. Connectivity of these two pathways is further supported by the observation that the presence of nitrate reduces the expression of genes involved in tetrathionate respiration as well as the growth advantage conferred by tetrathionate respiration (Lopez et al., 2012). It is currently unclear how *S*. Tm balances the sensing and utilization of two signals that may restrict one another.

Much of our understanding of the host–microbiota–pathogen interplay has been generated through genetic manipulation of the pathogen, and alternative experimental approaches are shedding new light on this interaction. For example, defined commensal communities have been used to reconstitute the microbiota of germ-free mice and study the contributions of specific members of the microbiota necessary for effective colonization resistance (Dewhirst et al., 1999; Stecher et al., 2010; Brugiroux et al., 2016). Studies using defined microbial communities will also contribute to understanding how differences in microbial species between susceptible and resistant intestinal environments impact *S.* Tm virulence gene expression. Additionally, a recent RNA-seq study assessed gene expression in *S*. Tm grown under 22 different *in vitro* conditions that mimicked aspects of infection, which could reveal new signals important for controlling the expression of key metabolic and virulence determinants (Kröger et al., 2013). Future *in vivo* metabolomics studies may identify specific microbiota or diet-dependent molecules that impact *S.* Tm virulence gene expression and/or host response. In line with this, a high salt diet was recently identified as significantly altering the host response during *S.* Tm infection (Tubbs et al., 2017). Thus, the ability to manipulate commensal microbial communities and intestinal metabolite concentrations offers an exciting tool to further understand how microbiota and metabolites not only impact *S*. Tm growth but also impact *S*. Tm virulence.

Collectively, these findings highlight that *S.* Tm thrives during host dysbiosis, benefits from the host immune response, and utilizes virulence factors to directly and indirectly suppress members of the microbiota. However, *S.* Tm relies on members of the indigenous microbial community to expand its metabolic capabilities during infection that in turn promote outgrowth and transmission (Maier et al., 2013). Although some host factors and cytokines that are involved in the development of inflammation during infection are known (Behnsen et al., 2015), additional components are likely to contribute to *S.* Tm infection. A more comprehensive understanding of immune responses that contribute to dysbiosis and/or generate a nutritional niche for *S.* Tm is necessary to fully understand *S.* Tm infection strategies, and indeed, this remains an active area of research. Host inflammatory components could be enhancing intestinal pathology, restricting microbiota reconstitution, molding a new nutrient niche, or enhancing all aspects of infection.

## Intramacrophage Adaptation

After benefiting from components of the host immune response during intestinal colonization, *S.* Tm must withstand the bactericidal efforts of host phagocytes during systemic infection. *S.* Tm dissemination from the intestinal tract to systemic sites of infection largely depends on phagocytic cells (Vazquez-Torres et al., 1999). Of the host phagocytes, macrophages frequently interact with *S.* Tm during dissemination and within systemic sites, including the spleen and liver (Salcedo et al., 2001). Macrophages utilize several strategies such as acidification of phagosomes, generation of reactive oxygen and nitrogen species, and production of antimicrobial proteins and peptides to kill internalized pathogens (Flannagan et al., 2009); however, *S.* Tm is able to withstand these defense mechanisms through multiple molecular mechanisms. The T3SS-2 and associated secreted effectors create a replicative niche within macrophages termed the *Salmonella* containing vacuole (SCV) by modulating diverse host processes (Hensel et al., 1995; Ochman et al., 1996; Shea et al., 1996; Figueira and Holden, 2012). For example, these effectors inhibit SCV–lysosome fusion, modify host vesicle trafficking, localize the SCV within the cell, and evade the host autophagic response (Uchiya et al., 1999; Shotland et al., 2003; Guignot et al., 2004; Boucrot et al., 2005; Brumell and Scidmore, 2007; Jackson et al., 2008; Owen et al., 2014, 2016). Multiple environmental signals and bacterial regulators influence this critical adaptation step (discussed below).

## Counteracting Host Defense Mechanisms

Pathogen recognition is the first line of defense for phagocytic cells such as macrophages. Host Toll-like receptors (TLRs) are transmembrane proteins located on the plasma membrane and endosomal membranes that recognize conserved molecular patterns associated with pathogens such as lipids, proteins, and nucleic acids (Kawai and Akira, 2010). TLRs have profound impacts on adaptive immunity and are thus broadly essential for host defense (Iwasaki and Medzhitov, 2004). Additionally, TLRs are critical for control of *S.* Tm replication as well as host survival during infection (Weiss et al., 2004). TLR activation and downstream signaling through the adapter proteins MyD88 and TRIF is linked to initial SCV acidification (Arpaia et al., 2011). The SCV acidifies rapidly following phagocytosis, dropping to a pH between 4.0 and 5.0 within 60 min of formation (Rathman et al., 1996). Acidification of the SCV is essential for *S.* Tm expression and functional formation of the T3SS-2 as well as secretion of effectors (Rathman et al., 1996; Beuzón et al., 1999; Hansen-Wester et al., 2002; Rappl et al., 2003; Arpaia et al., 2011; Chakraborty et al., 2015). Thus, *S.* Tm co-opts part of the macrophage defense system to serve as an initiating signal that promotes bacterial survival.

In the host, concentrations of free iron are extremely low in part to limit growth of invading pathogens (Kühn, 2015; Behnsen and Raffatellu, 2016). For example, the host expresses iron regulatory proteins (IRPs) and lipocalin-2 to limit the amount of iron available within macrophages (Kühn, 2015; Nairz et al., 2015a,b). Host-mediated iron limitation significantly restricts *S.* Tm replication within macrophages and reduces lethal disease (Nairz et al., 2015a). Additionally, IRP and lipocalin-2 influence the immune response during *S.* Tm infection (Nairz et al., 2015a,b). Indeed, modulating the immune response, specifically interleukin 10, can rescue the bactericidal defects of lipocalin-2 deficient cells and mice (Nairz et al., 2015b). These results suggest that host iron concentrations indirectly affect *S.* Tm survival through alteration of the host immune response rather than by directly starving *S.* Tm of iron. Nonetheless, modulation of iron concentrations does influence *S.* Tm expression of virulence genes. For example, SPI-2 expression is reduced when *S.* Tm is grown in the presence of iron (Choi and Groisman, 2013; Choi et al., 2014), and the iron-sensing transcription factors Fur and PmrA limit SPI-2 expression during macrophage infection (Wösten et al., 2000; Choi and Groisman, 2013; Choi et al., 2014). However, SPI-2 expression has also been shown to be reduced when iron is chelated from cultures and during infection of macrophages that have low iron concentrations (Zaharik et al., 2002; Nairz et al., 2009). These contrasting findings require further investigation. Similarly, it is unclear what the iron availability is within the SCV with and without host iron acquisition mediators IRP and lipocalin-2.

Altogether, these findings highlight that *S.* Tm senses and responds to macrophage defense mechanisms, which impact *S.* Tm virulence and survival. By utilizing host defenses as signals, *S.* Tm incorporates antimicrobial processes into a bacterial signal transduction pathway that creates a suitable replication niche in an otherwise inhospitable environment.

## Intramacrophage Sensing and Signaling

**FIGURE 2:**
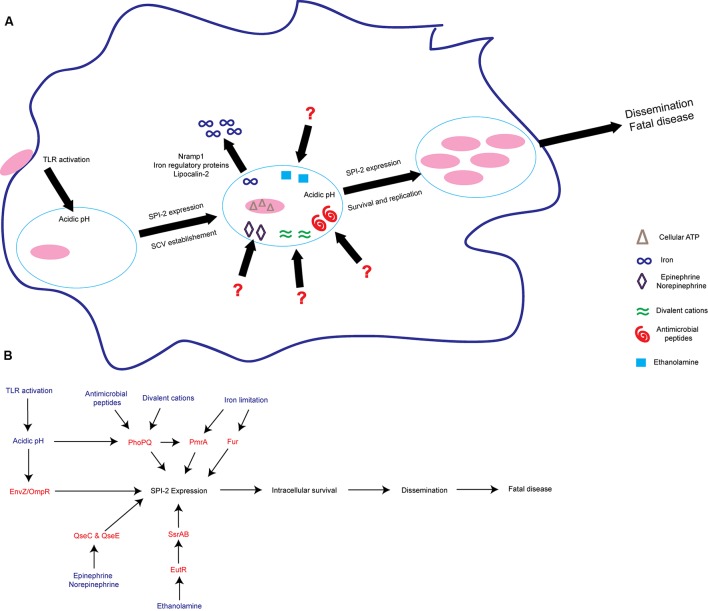
*S.* Tm senses environmental cues to adapt to the intramacrophage environment. **(A)** *S.* Tm recognizes diverse signals within the macrophage. These include iron limitation, ethanolamine, acidic pH, antimicrobial peptides, divalent cations, epinephrine/norepinephrine, and cellular ATP. These signals act to induce SPI-2 expression and establish the SCV as a replicative niche. **(B)** Schematic of some of the regulatory proteins and associated signals important for activating SPI-2 expression. Signals are written blue; regulatory proteins and TCS are written in red. TLRs, Toll-like receptors; SCV, *Salmonella* containing vacuole.

In addition to host defense-linked signals, *S.* Tm responds to concentrations of cations, nutrients, and ATP to activate expression of SPI-2 and other virulence factors to ensure survival (Valdivia and Falkow, 1997; Cirillo et al., 1998; Deiwick et al., 1999; Kim and Falkow, 2004; Löber et al., 2006; Osborne and Coombes, 2011; Lee and Groisman, 2012; Blair et al., 2013) (**Figure 2**). To sense these environmental signals and regulate SPI-2 expression, *S.* Tm uses several two-component systems (TCS) (Fass and Groisman, 2009). TCS are typically comprised of a sensor kinase that autophosphorylates upon sensing of a stimulus and then phosphorylates its paired response regulator, which in turn binds DNA to activate transcription of target genes (Stock et al., 2000). The PhoPQ TCS senses acidic pH, divalent cations, antimicrobial peptides, and potentially other signals to activate SPI-2 expression and modify components of the bacterial outer membrane (Bader et al., 2005; Prost et al., 2007; Dalebroux et al., 2014; Hicks et al., 2015). In addition to SPI-2, PhoP regulates other virulence factors, including the *mgtCBR* operon. The *mgtCBR* operon encodes an inner membrane protein, a Mg^2+^ transporter, and a regulator and is critical for intramacrophage survival (Soncini et al., 1996; Blanc-Potard and Groisman, 1997; Alix and Blanc-Potard, 2007; Lee and Groisman, 2010). It is still unclear what host or bacterial factors contribute to the presence of these SPI-2 activating signals within the SCV.

*S.* Tm also responds to signals that are not specific to the SCV, but rather are found in multiple environments throughout the host. For example, the host hormones epinephrine and norepinephrine (epi/NE) are ubiquitous throughout the body (Boyanova, 2017). The bacterial TCS QseBC and QseEF sense and respond to epi/NE during infection (Clarke et al., 2006; Reading et al., 2009). The sensor kinase QseC regulates the expression of genes encoded within SPI-1 and enhances epithelial cell invasion under conditions that promote SPI-1 expression (Moreira et al., 2010). Additionally, during macrophage infection, QseC activates SPI-2 expression to enhance intramacrophage survival (Moreira et al., 2010). Moreover, both epi/NE-responsive histidine sensor kinases, QseC and QseE, are required for systemic infection (Rasko et al., 2008; Moreira et al., 2010; Moreira and Sperandio, 2012). NE induces expression of both SPI-1 and SPI-2 associated genes, however, only expression of SPI-2 associated genes is QseC dependent (Moreira et al., 2010). These findings reveal that the same signal (epi or NE) enhances *S.* Tm virulence gene expression depending on the surrounding environment. The additional components of the intramacrophage environment that allow these epi/NE-dependent signaling pathways to distinguish between a SPI-1 or SPI-2 inducing condition warrant further studies.

Ethanolamine is another signal that plays environment-dependent roles in expression of *S.* Tm virulence traits (Anderson and Kendall, 2016). Ethanolamine is present in serum and is maintained intracellularly by host cells in part to recycle and produce phosphatidylethanolamine (Nikawa et al., 1986; Lipton et al., 1988, 1990; Sandra and Cai, 1991; Shiao and Vance, 1995). In the Enterobacteriaceae, including *S.* Tm, the transcription factor EutR directly senses ethanolamine (Roof and Roth, 1992; Luzader et al., 2013). EutR-dependent signaling promotes ethanolamine metabolism during intestinal infection (Anderson et al., 2015). As infection progresses, ethanolamine promotes *S*. Tm dissemination to systemic sites independently of metabolism (Anderson et al., 2015). Although ethanolamine metabolism does not provide a growth benefit for *S.* Tm during systemic infection (Stojiljkovic et al., 1995; Thiennimitr et al., 2011; Steeb et al., 2013; Anderson et al., 2015), EutR directly activates expression of SPI-2 within macrophages leading to increased survival and early dissemination (Anderson et al., 2015). It is currently unclear how ethanolamine can signal through the same receptor, EutR, to promote niche adaptation in distinct host environments. However, it is clear that ethanolamine plays a dual role in *S*. Tm infection by supporting growth in the inflamed intestine as well as enhancing subsequent stages of *S*. Tm disease.

The production of *Salmonella*-induced filaments (SIFs) is a critical component of *S.* Tm adaptation to intracellular environments (Garcia-del Portillo et al., 1993; Stein et al., 1996). This SIF network remodels the host endosomal network, allowing *S.* Tm to gain access to endocytosed molecules (Ohlson et al., 2008; Liss et al., 2017). The SCV/SIF continuum is required for efficient intracellular metabolism and promotes *S.* Tm replication (Liss et al., 2017). While the focus of this study was on access to metabolites (Liss et al., 2017), the same principle of incorporating extracellular molecules into the SCV may be true for signaling molecules that promote virulence. In such a model, signaling molecules present within host endosomes, or recently endocytosed molecules from the extracellular environment, would be shuttled through the SCV/SIF continuum and potentially impact *S*. Tm virulence gene expression. This revelation opens the possibility that large sets of molecules within various host tissues are able to reach the SCV within macrophages *in vivo*. Additionally, genes that have previously been identified as not being induced during macrophage infection *in vitro* may be the result of a relevant signal being absent from the culture conditions. Determining *in vivo* signals that *S.* Tm recognizes remains a daunting challenge but is necessary for a thorough understanding of the events that trigger intracellular adaptation.

The majority of work on environmental signals and sensing within macrophages has focused on transcriptional regulation; however, recent proteomic and RNA-seq approaches have shown that regulation of gene expression is more complex. Post-translational modifications as well as small regulatory RNA (sRNA)-induced post-transcriptional changes regulate virulence during macrophage infection (Ansong et al., 2013; Westermann et al., 2016). sRNAs are non-coding 50–500 nucleotide transcripts that utilize base-pair interactions to post-transcriptionally regulate the expression of target mRNAs (Hébrard et al., 2012). *S.* Tm encodes approximately 300 unique sRNAs, and the expression of a subset of sRNAs is highly sensitive to signals encountered within the macrophage environment, such as nutrient starvation, and are controlled by key components of the SPI-2 regulatory system (Kröger et al., 2012, 2013; Amin et al., 2016; Colgan et al., 2016). Specifically, the sRNA PinT regulates SPI-2 expression and is critical for host adaptation *in vivo* (Chaudhuri et al., 2013; Westermann et al., 2016). It is possible that post-transcriptional regulation helps *S.* Tm incorporate environmental signals sensed within the SCV to modulate survival.

In addition to active intracellular replication, entering a non-replicating yet viable state is an alternative adaptation strategy. A portion of the infecting *S.* Tm population enters a persistent state within macrophages that is independent of SPI-2 (Helaine et al., 2010). These non-replicating *S.* Tm remain viable and are not killed by macrophage antimicrobial defenses (Helaine et al., 2010). This phenomenon has also been demonstrated within non-phagocytic cells *in vivo* (Núñez-Hernández et al., 2013). It is unclear how these persister cells impact disease progression *in vivo*, but perhaps persister cells promote asymptomatic carriage and transmission. Future studies are required to determine if there is transcriptional overlap between these persister cells and actively replicating *S.* Tm or if perhaps post-transcriptional regulation contributes to this phenotypic switch. Advancements in single cell expression techniques will allow replicating and dormant cells to be distinguished from one another. Similarly, it remains unclear what signals present within the SCV trigger the shift from replication to persistence. Although distinct signals may be responsible for transitioning to a persistent state, it is also possible that loss of the signals important for replication leads to dormancy.

Altogether, these studies reveal complex and dynamic regulatory circuits important for *S*. Tm survival within macrophages. *S.* Tm must appropriately repress and activate virulence gene expression to ensure adaptation to the SCV (Kato et al., 2003; Choi and Groisman, 2013; Westermann et al., 2016). Additionally, *S.* Tm must be able to initiate distinct regulatory pathways at different time points during infection (Moreira et al., 2010; Moreira and Sperandio, 2012; Anderson et al., 2015). Further understanding of these activating and suppressing cues, and how they are balanced with one another, will enhance our understanding of *S.* Tm pathogenesis.

## Host Cell Death

The replication-suitable SCV within macrophages is a temporary niche, as host cells die. The way in which cells die has a tremendous impact on host physiology and inflammation (Pasparakis and Vandenabeele, 2015; Medina and Ravichandran, 2016). Programmed host cell death can be classified based on the effector proteins required and the state of inflammation each type of death induces (Kroemer et al., 2009). Three of the major programmed cell death pathways are apoptosis, necroptosis, and pyroptosis (Kroemer et al., 2009). *S.* Tm infection induces apoptosis-like features in infected macrophages including chromatin condensation and caspase-3 and caspase-8 activity (Chen et al., 1996; Lindgren et al., 1996; Monack et al., 1996; van der Velden et al., 2000; Man et al., 2013; Günster et al., 2017). Additionally, *S.* Tm infection can induce RIPK3 and MLKL-dependent necroptosis (Robinson et al., 2012; Günster et al., 2017). The best-characterized form of *S.* Tm-induced cell death is caspase-1/caspase-11-dependent pyroptosis (Brennan and Cookson, 2000; Monack et al., 2001; Fink and Cookson, 2006; Fink et al., 2008). Host cells employ several means to recognize *S.* Tm virulence proteins and initiate regulated pyroptosis. For example, macrophage NLRC4 (Ipaf) recognizes *S.* Tm flagellin and components of T3SS-1 to activate caspase-1 (Franchi et al., 2006; Miao et al., 2006, 2010b; Zhao et al., 2011). Additionally, macrophage NAIP1 also recognizes components of T3SS-1 to initiate a pyroptotic death (Rayamajhi et al., 2013; Yang et al., 2013). Caspase-1 and caspase-11, as well as their activators NLRP3 and NLRC4, impact *S.* Tm pathogenesis and bacterial burden *in vivo* (Lara-Tejero et al., 2006; Raupach et al., 2006; Broz et al., 2010, 2012). Interestingly, the host is able to enhance clearance of *S.* Tm infection when pyroptosis is experimentally induced *in vivo* (Miao et al., 2010a; Stewart et al., 2011; Aachoui et al., 2013; Jorgensen et al., 2016). These findings suggest that *S.* Tm might try to evade inducing host cell death during some stages of infection. Although most commonly studied in macrophages, *S.* Tm-induced cell death also occurs in dendritic and epithelial cells (van der Velden et al., 2003; Sellin et al., 2014; Rauch et al., 2017). It remains uncertain if infected cells are simply overwhelmed by *S.* Tm over time *in vitro*, if *S.* Tm actively regulates virulence to promote or evade host cell death *in vivo*, if cell death is occurring throughout infection, and what the consequences of host cell death are during systemic disease.

## Conclusion

*S.* Tm is able to recognize, adapt to, and survive within the intestinal tract and the intramacrophage environment during infection. These two environments present very different obstacles to infection, and *S.* Tm utilizes diverse strategies to overcome these distinct barriers. A better understanding of the signaling molecules, the signal transduction pathways, and the cross talk between signal transduction pathways that promote niche recognition may provide novel opportunities for therapeutic intervention. Importantly, several of the strategies used by *S.* Tm to adapt to host environments are conserved among several organisms such as pathogenic *E. coli*, *Listeria monocytogenes*, *Brucella abortus*, and *Staphylococcus aureus*. For example, pathogens adapt to the host or regulate virulence by utilizing and/or sensing nitrate (Spees et al., 2013; Winter et al., 2013), epi/NE (Curtis et al., 2014; Halang et al., 2015; Moreira et al., 2016; Rooks et al., 2017), ethanolamine (Maadani et al., 2007; Kendall et al., 2012; DebRoy et al., 2014; Gonyar and Kendall, 2014; Mellin et al., 2014; Subashchandrabose et al., 2014), iron (Almirón et al., 2001; Zimbler et al., 2012; Hammer et al., 2013, 2014; Mashruwala et al., 2015; Palmer and Skaar, 2016), and pH (Heinzen et al., 1996; Sturgill-Koszycki and Swanson, 2000; Singh et al., 2008; Vandal et al., 2008). Additionally, T6SS are widely conserved, particularly within Proteobacteria and Bacteroidetes (Hood et al., 2017), and promote colonization of intestinal pathogens (Fu et al., 2013; Sana et al., 2017). Perhaps the fact that *S.* Tm utilizes all of these strategies, rather than a select few, makes *S.* Tm so successful and such a major burden on global healthcare (Scallan et al., 2011). Therefore, further study of these environmental adaptation strategies, using *S.* Tm as a model organism, will enhance our understanding of the host–microbiota–pathogen interface.

## Author Contributions

All authors listed have made a substantial, direct and intellectual contribution to the work, and approved it for publication.

## Conflict of Interest Statement

The authors declare that the research was conducted in the absence of any commercial or financial relationships that could be construed as a potential conflict of interest.
